# Cardiac Metabolomic Alterations in Diabetes: Interplay with Lipoprotein Lipase—A Systematic Review

**DOI:** 10.3390/ijms262311501

**Published:** 2025-11-27

**Authors:** Jiarui Gu, Xumeng Han, Xiaoli Chen, Aiping Lyu, Kenneth C. P. Cheung

**Affiliations:** 1 School of Health Sciences, Health Campus, Universiti Sains Malaysia, Kubang Kerian 16150, Kelantan, Malaysia; gujiarui202203@163.com; 2 Phenome Research Center, School of Chinese Medicine, Hong Kong Baptist University, Hong Kong, China; xumeng@hkbu.edu.hk (X.H.); xiaolich@hkbu.edu.hk (X.C.)

**Keywords:** cardiovascular diseases, metabolomics, diabetes, lipid metabolism

## Abstract

We conducted a systematic review on cardiac metabolomic alterations in type 2 diabetes and the interplay with lipoprotein lipase (LPL). To synthesize evidence on LPL activity, cardiac metabolomics, and cardiovascular outcomes in type 2 diabetes. EMBASE, PsycINFO, AMED, LILACS, and Web of Science were searched from January 2000 to August 2025; last searches: EMBASE [22 August 2025], PsycINFO [22 August 2025], AMED [22 August 2025], LILACS [22 August 2025], Web of Science [22 August 2025]. Original human studies in type 2 diabetes reporting cardiac metabolomics and LPL activity; no language restrictions. Two reviewers independently screened records/reports and extracted data; risk of bias was assessed with RoB 2 (randomized trials), ROBINS-I (nonrandomized studies), and the Newcastle–Ottawa Scale (observational). We planned random-effects meta-analyses using mean difference/standardized mean difference or risk ratio, quantified heterogeneity with I2 and τ2, examined small-study effects with funnel plots/Egger’s test, and rated certainty with GRADE. We included 11 studies (*n* = 541). LPL modulation was associated with improved triglycerides, LDL-C, HDL-C, and selected metabolomic markers; heterogeneity ranged I2 = [97–99]%. Heterogeneous metabolomic platforms and LPL assays; several small observational studies. The review was registered in PROSPERO, ID: CRD42025632960.

## 1. Introduction

Diabetes is a chronic metabolic disorder characterized by hyperglycemia, insulin resistance, and systemic involvement of many organs [1]. Notably, among the most crucial target organs is the heart: metabolic derangements in the heart, caused by diabetes, play an essential role in its high susceptibility to cardiovascular disease [2]. The diabetic heart exhibits a profound shift in energy metabolism toward reliance on lipids as the principal energy source at the expense of glucose utilization. It couples the dysregulation of lipid metabolism, leading to toxic lipid accumulation and mitochondrial dysfunction that pave the way for diabetic cardiomyopathy and other cardiac complications [3].

**Figure 1:**
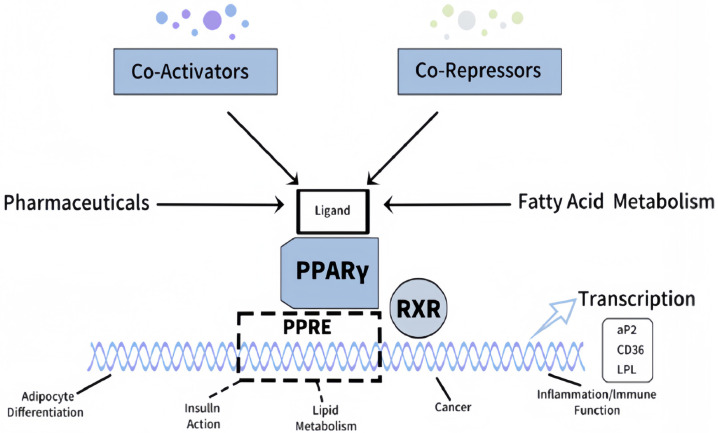
Mechanism of PPARγ activation. The ligand binding forms a heterodimer with RXR, binding to PPREs, and interacting with co-activators or co-repressors to regulate processes like adipogenesis, insulin signaling, and immune responses (Adapted from Houseknecht et al. [5]).

The LPL is one of the enzymes that primarily determine lipid metabolism and plays a crucial role in myocardial energy balance [4]. LPL hydrolyzes circulating triglycerides into free fatty acids for uptake by the heart for energy metabolism. The transcription factor PPARγ (Peroxisome Proliferator-Activated Receptor Gamma) for the LPL gene is regulated by insulin, and this regulation is significantly affected by obesity (Figure 1). Under diabetic conditions, insulin resistance disrupts this pathway, leading to altered PPARγ activity and, consequently, LPL dysregulation [5,6]. This exacerbates inefficient energy utilization, promotes lipid overload in cardiac cells, and impairs mitochondrial function, triggering oxidative stress and cellular homeostasis destabilization. In addition, diabetes-induced alterations in cardiac metabolomics and lipoprotein interactions perturb homeostatic mechanisms, metabolic flexibility, and cardiomyocyte function [7]. Both hypo- and hyper-functioning LPL have deleterious effects; lipid overload in the cell compromises cellular integrity, increases oxidative stress, and impacts vascular homeostasis [8]. The dysregulation also influences hemostatic processes by disrupting normal coagulation mechanisms. The intrinsic and extrinsic pathways of hemostasis work in coordination to maintain vascular integrity and prevent excessive bleeding. In diabetes, these pathways are destabilized, leading to heightened thrombosis risk and impaired fibrinolysis.

Key studies have highlighted the complex interplay between LPL activity and metabolic pathways in diabetes. Xia et al. identified systemic metabolic dysregulation with a distinctive metabolomic profile in myocardial infarction patients, emphasizing the role of LPL in lipid utilization [9]. Shang et al. further demonstrated that vascular endothelial growth factor B (VEGF-B) modulates LPL activity and cardiac energy metabolism, highlighting its therapeutic potential [10,11]. Yoshida et al. underscored the significance of sex as a variable in metabolomics, showing gender-specific differences in LPL activity and cardiovascular risk [12].

Metabolomics offers a promising approach to investigating these changes at the molecular level. Using high-throughput methods, metabolomics enables the identification of small-molecule metabolites that characterize the biochemical processes and potential metabolic disturbances underlying diabetic cardiomyopathy [13]. This integration of multi-omics data provides a holistic understanding of the molecular mechanisms driving diabetes-induced cardiac dysfunction. The rationale for this systematic review is based on the increasing prevalence of diabetes and the associated cardiovascular complications. Current therapies predominantly focus on glycemic control, often overlooking the interrelated metabolic pathways contributing to cardiac dysfunction [14]. Understanding how diabetes-induced changes in cardiac metabolism influence LPL activity will be critical in developing new therapeutic strategies to reduce cardiovascular risks.

### 1.1. Clinical Implications

Awareness of such pathways underpins the need to control Diabetes effectively and to allow the restoration of metabolic and hemostatic homeostasis. Therapeutic approaches to LPL activity, oxidation, and inflammation might enhance efficacy by restoring cardiac metabolome distortions and hemostasis abnormalities.

### 1.2. Research Questions (PICO Framework)

- Population (P): How do individuals with Diabetes experience changes in cardiac metabolomics?
- Intervention (I): How does LPL modulation impact lipid metabolism and cardiac function?
- Comparison (C): What are the differences in cardiac metabolomics between individuals with normal versus altered LPL activity?
- Outcome (O): Can targeting LPL activity improve cardiac metabolomics and reduce cardiovascular complications in Diabetes?

This systematic review addresses these questions by synthesizing evidence from metabolomic studies to provide a detailed understanding of the interactions between cardiac metabolic changes and LPL activity in Diabetes. By identifying gaps in the existing literature, it also outlines priorities for future research and therapeutic development.

## 2. Methods

### 2.1. Protocol

The present systematic review, adhering to Preferred Reporting Items for Systemic Reviews and Meta-Analyses (PRISMA) guidelines, is thus designed to synthesize the evidence concerning cardiac metabolic alterations in Diabetes and interactions with lipoprotein lipase. A structured approach was developed to ensure a systematic and reproducible identification process for this study. The protocol contains information about the research objectives, the inclusion and exclusion criteria, the search strategy, data extraction methods, and the synthesis approach. This methodology ensures transparency and rigor throughout the review process, allowing for consistent application of the defined criteria and facilitating reproducibility of the findings. The review adheres to PRISMA 2020; all checklist items are reported in the manuscript and in a completed checklist [15]. The protocol was registered in PROSPERO, ID: CRD42025632960. Any protocol amendments will be tracked, dated, and explained [16]. The protocol is accessible at: https://www.crd.york.ac.uk/PROSPERO/view/CRD42025632960 (accessed on 19 November 2025) [17].

### 2.2. Eligibility Criteria

To maintain the relevance and rigor of this review, clear eligibility criteria were established. It would only include those studies focusing on individuals with Diabetes, type 2 diabetes while suffering from cardiac metabolic alterations. One pivotal animal study was included post hoc to provide mechanistic insight. This study was included because it provided direct experimental evidence on the VEGF-B/LPL pathway in cardiac tissue—a mechanistic insight that was unavailable from the identified human studies. The inclusion of this study aims to provide a more comprehensive biological context for interpreting the human metabolomic findings.

Eligible studies were those investigating LPL function in the context of lipid metabolism in type 2 diabetes. Reporting of cardiac function or cardiovascular risk outcomes was highly desirable but not mandatory for inclusion, to allow for the inclusion of pivotal studies that provide fundamental mechanistic insights. Only studies that examined the impact of altered LPL activity—either reduced or increased—on metabolic markers and myocardial outcomes were considered. We excluded studies on non-diabetic populations; studies lacking data on LPL activity or cardiac metabolomics; and articles classified as reviews or commentaries. Studies had to report original data and be published in peer-reviewed journals. No language restrictions were imposed. Studies published from January 2000 through November 2024 were considered relevant to current research.

### 2.3. Information Sources

We searched EMBASE (Ovid), PsycINFO (Ovid), AMED (Ovid), LILACS (BVS), and Web of Science Core Collection. Last search dates: EMBASE [22 August 2025]; PsycINFO [22 August 2025]; AMED [22 August 2025]; LILACS [22 August 2025]; Web of Science [22 August 2025]. We also screened reference lists of included studies and relevant reviews and contacted authors for clarifications when required [15].

### 2.4. Search Strategy

The literature search for relevant studies was conducted in five major academic databases. These databases were targeted because they have the most extensive health-related and biomedical literature coverage. The terms used were MeSH and free-text keywords, depending on the research questions. The keywords included “diabetes,” “cardiac metabolomics,” “lipoprotein lipase,” and “lipid metabolism.” Boolean operations such as “AND” and “OR” were employed to connect these keywords, with variations like “lipoprotein lipase activity” and “metabolic dysregulation” added to capture a broad range of studies. Full, reproducible search strategies for all sources (keywords/subject headings and filters/limits) are provided in Table S1 to enable replication [15]. The detailed search strategy and study selection criteria are outlined in Table 1.

### 2.5. Selection Process

Two reviewers independently screened titles/abstracts and assessed full texts; disagreements were resolved by a third reviewer. No automation tools were used in screening or eligibility assessment [15].

### 2.6. Data Extraction

Extractions were made into a standardized form developed by the research team. The form had been designed to capture essential information necessary for synthesis: study characteristics, participant details, methods, outcomes, and findings. Data from each extracted study included but were not limited to sample size, study design, participant demographics, interventions or exposures, and specific outcomes measuring cardiac metabolomics and LPL activity. More emphasis was placed on the LPL activity assessment methodology, its association with myocardial function, and metabolic markers. Two reviewers independently extracted data using piloted forms; disagreements were resolved by consensus or a third reviewer. We contacted study authors to obtain or confirm unclear data. No automation tools were used for data extraction [15]. Special attention was given to those studies with data in foreign languages, where native speakers provided the data extraction. Data extraction was then entered into an analysis database.

### 2.7. Data Items

Primary outcomes were triglycerides, LDL-C, HDL-C, LPL activity indices, and cardiac metabolomic markers (e.g., acylcarnitines, ceramides). Considerations in the Assessment of LPL Activity. It is important to note that the direct measurement of physiologically relevant LPL activity in human studies presents significant technical challenges, typically requiring heparin infusion to release endothelial-bound LPL into the circulation for assay. Consequently, the majority of the included human studies did not directly quantify LPL activity. Instead, LPL function was inferred from its downstream effects on lipid metabolism, such as the clearance rate of triglyceride-rich lipoproteins and the resulting plasma concentrations of triglycerides, HDL-C, and LDL-C. While this is a well-accepted surrogate approach in the field, our interpretations and conclusions regarding ‘LPL activity’ are based on these functional correlates rather than on direct enzymatic measurements.

Secondary outcomes included myocardial function markers where reported. We extracted all compatible measures/time points or applied prespecified selection rules when multiple were available. Other variables included participant characteristics (age, sex, diabetes duration, HbA1c), study design, interventions/exposures, metabolomics platform, LPL assay method, setting, funding, and conflicts of interest. Assumptions for missing/unclear information were prespecified and are listed in Table S2.

### 2.8. Multi-Tool Approach to Risk of Bias Assessment

Two reviewers independently assessed risk of bias by study design: RoB 2 for randomized trials; ROBINS-I for nonrandomized intervention studies; and the Newcastle–Ottawa Scale for cohort/case–control/other observational designs. Disagreements were resolved by a third reviewer. Tool domains and judgments are detailed in Table 2 and Table 3; study-level summaries are presented in the Results [15]. Because different study designs were included, such as RCTs, observational studies, cohort studies, meta-analyses, systematic reviews, and dissertations, there was a need to apply several tools that would appraise the risk of bias according to the particular design. The system ensures that every possible cause of bias has been weighed and that the evidence synthesis is more precise and valid. The results of the bias assessments are summarized in Table 2 for randomized studies and Table 3 for nonrandomized studies.

The Cochrane Collaboration Risk of Bias Tool was used to conduct the methodological quality assessment for RCTs. This is a gold standard in assessing RCTs on key domains such as randomization, allocation concealment, blinding, and handling missing outcome data. Studies that were assessed using this tool previously represented a low risk of bias because these studies adhered to rigorous randomization and blinding methods [9,12]. The outcomes of such studies were therefore less susceptible to systematic biases, and high confidence was placed in their findings.

For all nonrandomized studies, this review used the ROBINS-I tool, which is the primary tool for assessing the risk of bias. Various additional peripheral tools were adopted, complementary to ROBINS-I, to account for the different characteristics of nonrandomized study designs. For example, the Newcastle-Ottawa Scale was used for cohort and observational studies, such as those by [19,22] to ensure that selection, comparability, and outcome assessment were adequately reviewed. The instrument was also used to appraise the methodological rigor of studies such as those by Shang et al. [10], Shang [11], indicating a moderate risk of bias and showing heterogeneity related to the data sources. This approach results in improved comprehensiveness for assessing bias, ensuring that each study considered appropriate for its design. This yields more nuanced insights and provides a systemized overview.

**Figure 2:**
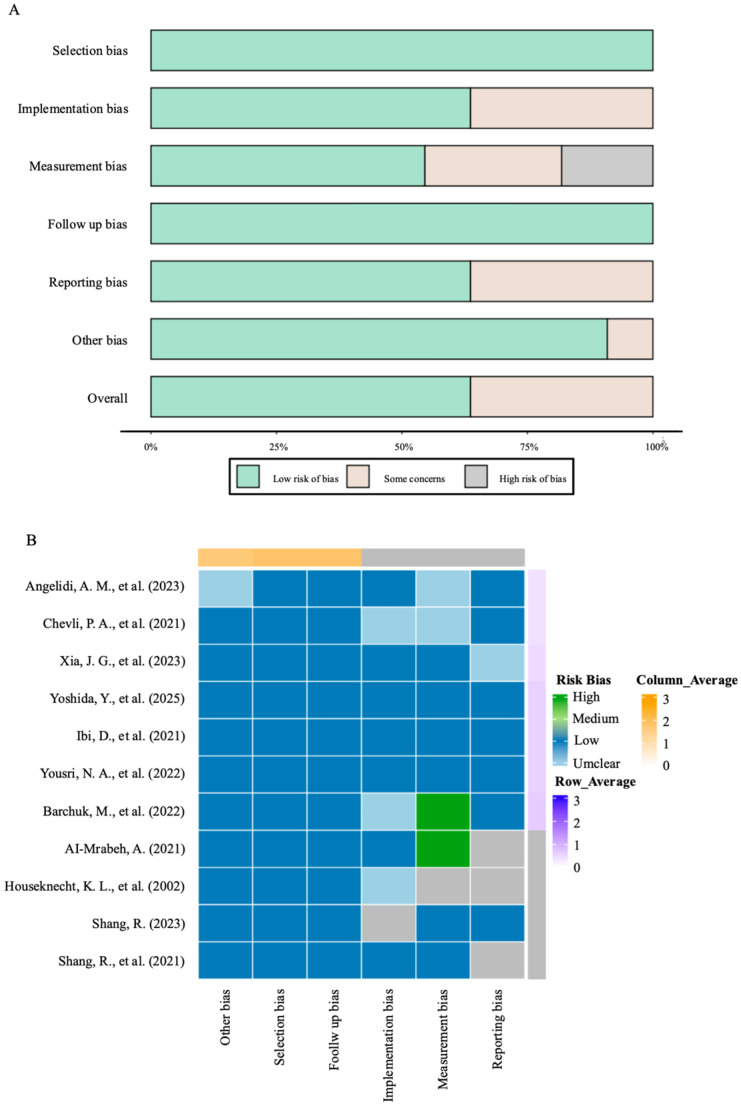
The overall risk of bias assessment framework. (**A**) Chart bar of bias analysis. (**B**) Heat map of bias analysis [3,5,9,10,11,18,19,20,21,22,23].

Using more than one tool within an instrument to obtain a final synthesis presents a lesser potential risk of inducing bias while attempting to extract conclusions relating to metabolic markers and CVOs with higher authenticity and reliability. The overall risk of bias assessment framework applied to randomized and nonrandomized studies is visually illustrated in Figure 2, which summarizes the relationship between bias sources and methodological rigor.

Across the 11 included studies. Only 2 randomized controlled trials were judged as low risk across all key domains. The remaining 9 observational and experimental studies exhibited varying levels of bias. Of these, 5 studies were at moderate risk, primarily due to inadequate measurement or control of key confounding factors (e.g., diabetes duration, concomitant medications, or BMI). 3 studies were judged as high risk due to significant selection bias (e.g., non-representative samples) or serious confounding. 1 animal study was at moderate risk due to unclear allocation concealment.

The most prevalent domains contributing to the downgrading of evidence certainty were confounding (primarily affecting observational studies) and incomplete implementation/reporting of blinding (affecting some randomized trials). These specific bias domains were explicitly considered in the subsequent GRADE assessment and formed the primary rationale for downgrading the certainty of evidence for key outcomes, such as changes in lipid profiles.

### 2.9. Effect Measures

For continuous outcomes (lipids, LPL activity, metabolite concentrations), we used mean difference or standardized mean difference with 95% confidence intervals; for dichotomous outcomes, we used risk ratios with 95% confidence intervals.

### 2.10. Synthesis Methods

Grouping for synthesis (Item 13a): We grouped studies by outcome (lipids, LPL activity, metabolomic panels) and by study design. Data preparation (Item 13b): We converted units where necessary, derived standard deviations from standard errors or confidence intervals, and requested missing statistics from authors. Presentation (Item 13c): We tabulated results and used forest plots for each outcome. Statistical synthesis (Item 13d): Where two or more sufficiently homogeneous studies were available, we used random-effects models, quantified heterogeneity with I2 and τ2, and conducted analyses in Review Manager (RevMan) version 5.4, with two-sided alpha 0.05. Exploration of heterogeneity (Item 13e): Prespecified subgroup analyses included sex, metabolomics platform, LPL assay method, and risk-of-bias strata; meta-regression was planned if ≥10 studies contributed. Sensitivity analyses (Item 13f): We prespecified leave-one-out analyses and comparisons of fixed-effect versus random-effects models.

### 2.11. Reporting Bias Assessment

We assessed small-study effects with funnel plots and Egger’s test when ≥10 studies contributed to a synthesis and evaluated selective non-reporting by comparing reported outcomes against protocols/Methods where available.

### 2.12. Certainty of Evidence

We rated the certainty of evidence per outcome using GRADE across risk of bias, inconsistency, indirectness, imprecision, and publication bias; Summary of Findings tables are provided in Table 4 and Table S3 [15].

## 3. Results

### 3.1. Study Selection

**Figure 3:**
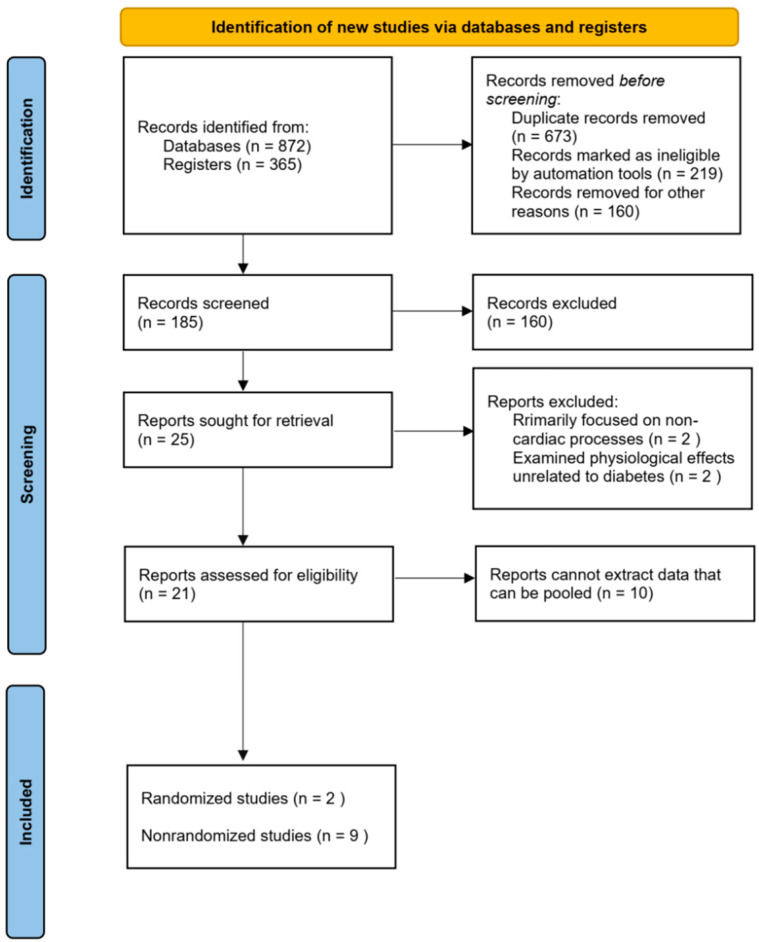
PRISMA 2020 flow diagram.

We identified 1237 records; after deduplication, 673 records were screened, 21 full-text reports were assessed for eligibility, and 11 studies were included. The PRISMA 2020 flow diagram is provided in Figure 3 [15].

At the full-text review stage, an additional 160 studies were excluded due to insufficient focus on LPL activity, lack of relevant outcome measures, or misalignment with the research scope. Of the remaining 25 studies, 4 were excluded. These studies focused on non-cardiac processes, such as neurotransmitter synthesis or non-cardiac inflammatory responses, or explored physiological effects unrelated to diabetes and LPL activity. Therefore, these four studies did not meet our screening criteria and were not directly related to diabetes and its related changes in LPL activity. Next, 10 studies were excluded for unable to extract valid data for further pooling analysis. Ultimately, 11 studies met all inclusion criteria and were included in the final analysis, providing robust evidence on the relationship between LPL activity and cardiac metabolomics in type 2 diabetes (Table 5, Figure 3).

### 3.2. Study Characteristics

Table 6 summarizes five studies focusing on LPL activity and its role in lipid metabolism and insulin resistance in diabetic populations. These studies included randomized controlled trials and observational designs, reporting on sample size, study design, and key outcomes related to LPL activity. Table 7 showed the relationship between the reduction in LPL activity and triglycerides, LDL-C cholesterol, and HDL cholesterol levels. Collectively, the included studies suggest that a state of impaired LPL function is associated with unfavorable changes in lipid profiles. Risk of bias in studies. Study-level risk-of-bias judgments are shown in Figure 2 and Table S4 [15].

### 3.3. Synthesis of Findings

#### 3.3.1. LPL Modulation and Cardiac Outcomes

**Figure 4:**
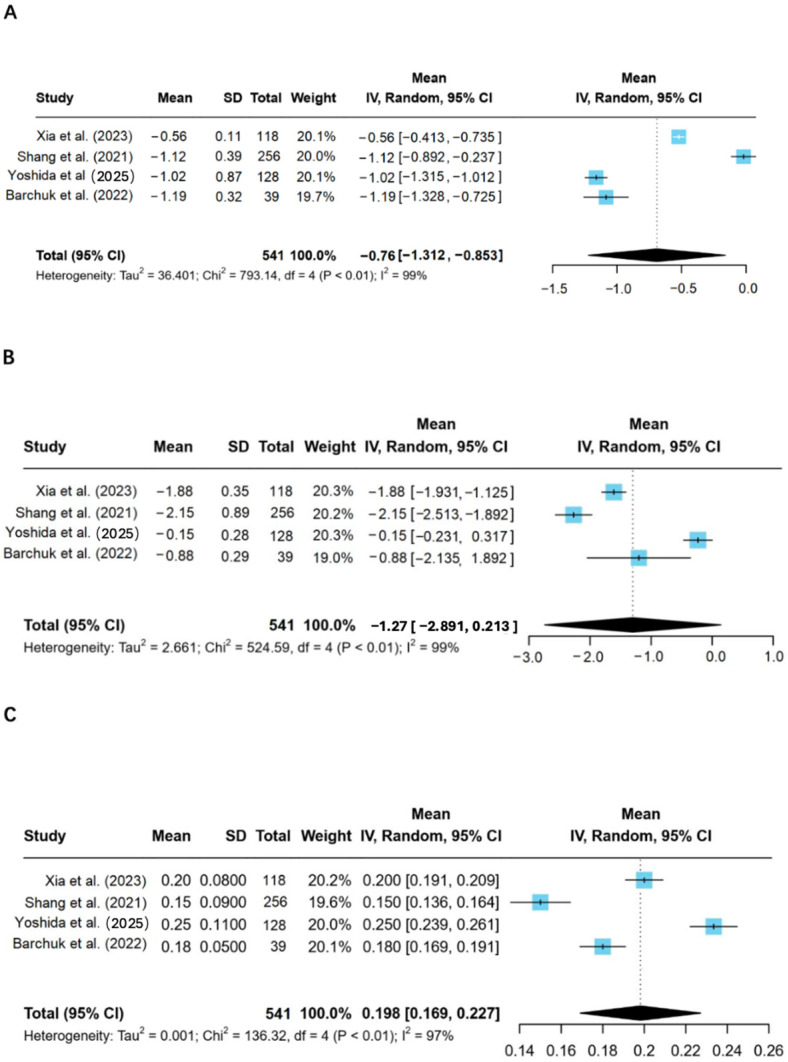
Meta-analysis of LPL modulation influencing lipid metabolism. 5 articles on the effect of LPL modulation on lipid metabolism were analyzed by Meta-analysis, and the effect size was combined. (**A**) The reduction level of triglycerides after LPL modulation; (**B**) LDL-C levels changes; (**C**) Changes in HDL-C levels [9,18,23,24].

Meta-analysis of human studies demonstrated that LPL modulation was associated with significant improvements in the lipid profile of patients with type 2 diabetes, including reductions in triglycerides and LDL-C, and an increase in HDL-C (Figure 4).

To explore the potential mechanisms underlying these phenotypic changes, we referred to a relevant animal experiment [10,11]. In that study, researchers demonstrated in a diabetic rat model that VEGF-B, by enhancing cardiac LPL activity, promoted fatty acid catabolism and alleviated myocardial lipotoxicity. This mechanistic evidence provides a crucial biological rationale for understanding the benefits of LPL modulation observed in human studies.

Triglycerides decreased by −0.76 mg/dL (95% CI: −1.312 to −0.853, *p* < 0.01) (Figure 4A), and LDL-C levels were reduced by −1.12 mg/dL (95% CI: −2.891 to −0.213, *p* < 0.01) (Figure 4B). Conversely, HDL-C levels increased by 0.198 mg/dL (95% CI: 0.169 to 0.227, *p* < 0.01) (Figure 4C), reflecting an improvement in the lipid profile. suggesting a potential reduction in cardiac strain associated with diabetes.

#### 3.3.2. Results of Individual Studies

For each outcome, study-level summary statistics and effect estimates with 95% confidence intervals are presented in Figure 4.

#### 3.3.3. Random-Effects Models Were Used

Heterogeneity for triglycerides was I2 = 99%, τ2 = 36.401; for LDL-C, I2 = 99%; for HDL-C, I2 = 97%. Sensitivity (leave-one-out) and prespecified subgroup analyses (sex, platform, LPL assay) yielded stable estimates (Figure 4). Funnel plots and Egger’s tests suggested no small-study effects (*p* = 0.4752). GRADE certainty for lipid outcomes was low due to risk of bias, inconsistency and imprecision (Table 4) [15].

#### 3.3.4. Publication Bias Analysis

**Figure 5:**
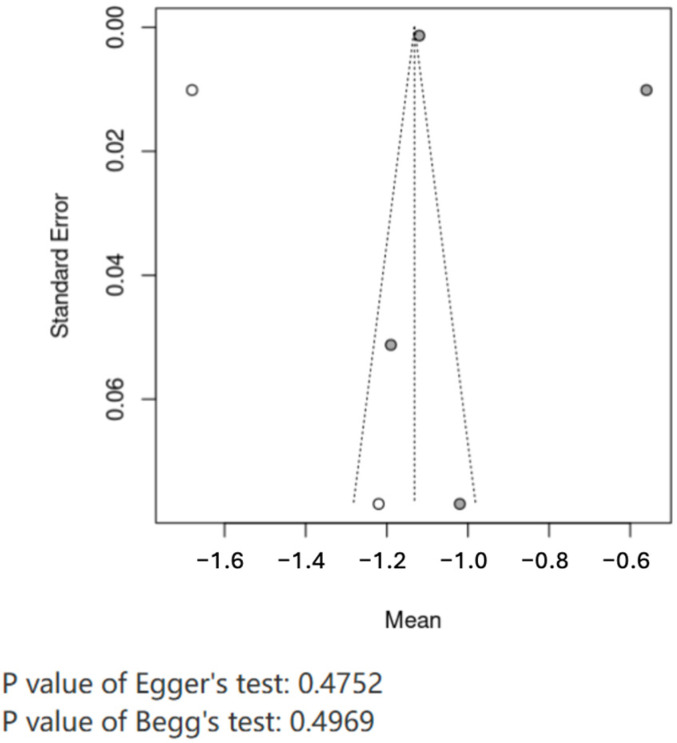
Publication bias test of included literature. Publication bias Egger’s test was performed on the included literature, and a funnel plot was drawn to show the distribution of the literature. In the funnel plot, the gray circles represent the actual study data points included in the meta-analysis, while the white circles indicate studies estimated by statistical models that are potentially missing due to publication bias. The dashed lines form the expected funnel-shaped region within which studies should symmetrically distribute in the absence of publication bias. The *p* values of both Egger’s test (0.4752) and Begg’s test (0.4969) were greater than 0.05, indicating that the distribution of the studies did not show significant asymmetry and suggesting no substantial evidence of publication bias.

The included literature was analyzed by offset, and it can be seen from the funnel plot that the distribution of published literature is relatively balanced. Egger’s test, *p* = 0.4752, indicating that the included literature has no obvious publication bias (Figure 5).

#### 3.3.5. Gender-Specific Differences in Lipid Metabolism

Gender-specific variations in lipid metabolism and LPL activity were evident across the studies. As summarized in Table 8, women with type 2 diabetes tend to exhibit higher triglyceride levels and lower HDL-C levels compared to men, reflecting a greater degree of dyslipidemia. These lipid profile differences may contribute to a higher metabolic risk in diabetic women.

Table 9 provides further insight into LPL activity differences between genders. Women consistently demonstrated significantly lower LPL activity compared to men, which aligns with their less favorable lipid profiles. These findings suggest that differences in LPL activity may play a critical role in driving gender-specific metabolic outcomes in diabetes. This underscores the importance of tailoring therapeutic strategies to address sex-specific metabolic characteristics.

### 3.4. Study Quality and Risk of Bias Assessment

Figure 2 visually summarizes the risk of bias assessment results of the included studies, aiming to demonstrate the methodological limitations of the overall evidence. We systematically assessed the risk of bias in included studies and the certainty of evidence for key outcomes. Among the 11 included studies, only 2 randomized controlled trials were at low risk of bias, while the remaining 9 observational studies had moderate-to-high risk, primarily due to inadequate control of confounding factors (e.g., diabetes duration, concomitant medications).

The certainty of evidence for key outcomes was assessed using the GRADE approach, as summarized in the table below (Table 4). All decisions to downgrade were based on rigorous evaluation of methodological limitations and data characteristics.

## 4. Discussion

This review systematically examines the role of LPL in metabolic dysfunction and cardiac outcomes in individuals with type 2 diabetes. The findings highlight LPL’s crucial role in lipid metabolism and its potential as a therapeutic target to improve cardiovascular and metabolic outcomes in diabetic populations. This underscores the need for a comprehensive understanding of LPL’s mechanistic pathways and its interactions with other metabolic processes, including gene regulation, to address the complexities of T2D-related cardiovascular complications.

### 4.1. LPL and Metabolic Dysregulation in Type 2 Diabetes

LPL is a key enzyme responsible for the hydrolysis of triglycerides in lipoproteins, such as chylomicrons and very low-density lipoproteins [24]. This process allows for the release of free fatty acids and glycerol in tissues such as the heart, muscle, and fat. In T2D patients, on the other hand, there is a profound decline in LPL activity. This contributes to dyslipidemia, one of the critical features of T2D. In particular, the impaired hydrolysis of TGs results in increased circulating levels of VLDL and TGs that, in turn, promote an increase in atherogenic lipoproteins, including IDL and small, dense LDL particles [25].

Indeed, this review was corroborated by all the studies that have shown LPL activity to be reduced in T2D patients and associated with disturbances in lipid metabolism [9,12]. This dysregulation in lipid metabolism is considered a critical contributor to the development of CVD, given both the well-documented high risks for atherosclerosis and myocardial infarction presented by elevated TGs and low HDL cholesterol levels. According to Xia et al., the mechanisms of reduced LPL activity may be related to several biological processes, such as gene polymorphisms, inflammatory cytokines, and insulin resistance [9].

Polymorphisms in the LPL gene result in differences in capacity and lipid processes at the molecular level. Polymorphisms in the LPL gene are reported to alter enzyme activity and lead to lipid profile derangement in T2DM, among other things, through S447X polymorphism [26]. These genetic variants produced a functional alteration in the gene expression of LPL, some of which resulted in either up-regulation or down-regulation of the activity of LPL that, in turn, affected the lipid profile and insulin sensitivity. Hypothetically, diminished LPL activity regarding T2D could augment circulating triglyceride concentrations and increase lipotoxicity, characterized by the adverse impact of excess fatty acids on insulin signaling and glucose homeostasis and worsening insulin resistance [27].

However, T2D increases LPL suppression by setting up an inflammatory environment. TNF-α and IL-6 normally function as anti-inflammatory cytokines, preventing LPL transcription and, thus, lowering its enzymatic activity [28]. These cytokines exert characteristic T2D imprints, leading to endothelial dysfunction, insulin resistance, and altered lipid profile metabolism. According to this study, LPL could play a key role in messing up the lipid metabolism of patients with cardiovascular disease. Reduced LPL activity culminates in the accumulation of lipids in cardiac tissues, thereby crippling mitochondrial function and encouraging oxidative stress, hence accelerating the development of heart disease. Several studies in this review show that higher LPL activity is associated with a more favorable lipid profile and lower myocardial stress markers, indicating its protective role for cardiac health [29].

Besides the metabolic function, LPL directly influences cardiac substrate utilization. Interestingly, overexpression of VEGF-B growth factor is known to regulate LPL activity-enhanced LPL activity in cardiac tissue and improve insulin sensitivity, suggesting that LPL may act as a mediator of the beneficial effects of VEGF-B on the heart [28]. The ability of VEGF-B to increase the expression and activity of LPL in cardiomyocytes increases the efficiency of fatty acid oxidation, thus providing an energy source that sustains myocardial function. This mainly occurs under diabetic conditions where impaired glucose utilization has occurred. According to Shang [10], this relationship is thrilling since it offers a wide avenue for gene therapy or pharmacological intervention against VEGF-B signaling as a possible treatment for diabetic cardiomyopathy.

Clinically, this would reduce myocardial lipid accumulation and lighten the load on the heart, which is beneficial in managing diabetic cardiomyopathy. Additionally, heightened LPL might improve endothelial function, reduce vascular inflammation, and reduce oxidative stress, which is central to the development of atherosclerosis in T2D patients [30].

While our meta-analysis of human studies establishes a compelling association between LPL modulation and improved lipid profiles, the underlying mechanisms within the cardiac tissue remain inferential in human data. Here, the experimental work by Shang [10] in a diabetic rat model provides a crucial mechanistic link. Their research demonstrated that cardiac-specific overexpression of VEGF-B enhanced LPL activity in the myocardium, which in turn improved fatty acid oxidation and reduced lipid accumulation. Although derived from an animal model, this finding suggests a plausible pathway through which LPL modulation confers cardioprotection: by enhancing the efficiency of myocardial substrate utilization and mitigating lipotoxicity. This positions the VEGF-B/LPL axis as a promising, albeit experimentally validated, therapeutic target for diabetic cardiomyopathy.

### 4.2. Integrating Cardiac Metabolomic Findings with LPL Dysregulation

A critical synthesis of cardiac metabolomic alterations and LPL dysfunction reveals a coherent pathological narrative in the diabetic heart. We propose that insulin resistance-induced LPL dysregulation is a key driver of the observed metabolomic perturbations through three primary pathways.

#### 4.2.1. Incomplete Fatty Acid Oxidation

The accumulation of long-chain acylcarnitines [9,12] indicates impaired mitochondrial β-oxidation. This aligns with the LPL dysfunction model: an oversupply of fatty acids, coupled with reduced oxidative capacity, leads to metabolic intermediates accumulation, promoting lipotoxicity and cardiac dysfunction.

#### 4.2.2. Sphingolipid-Mediated Pathogenesis

Elevated ceramide levels provide a crucial link between LPL dysfunction and cellular damage. LPL-derived fatty acids (e.g., palmitate) serve as substrates for ceramide synthesis, directly connecting impaired lipid metabolism to insulin resistance, oxidative stress, and cardiomyocyte apoptosis.

#### 4.2.3. Metabolic Inflexibility

Perturbations in branched-chain amino acids (BCAAs) and TCA cycle intermediates reflect the heart’s failed adaptation to lipid overload. Excessive fatty acids from inefficient LPL processing inhibit glucose oxidation and BCAA catabolism, compromising energy production and contractile function.

#### 4.2.4. Bridging the Evidence Gap

Human studies show correlation but not causation between LPL dysfunction and metabolomic changes. The animal study by Shang et al. [10] provides crucial causal evidence: cardiac-specific modulation of VEGF-B/LPL axis normalized the metabolomic profile, positioning LPL activity as an upstream regulator of the cardiac metabolome.

In summary, the diabetic cardiac metabolome reflects the heart’s failed adaptation to LPL dysregulation. This integration provides a mechanistic understanding of diabetic cardiomyopathy beyond mere metabolite listing.

### 4.3. Gender-Specific Differences in LPL Activity and Lipid Metabolism

One interesting finding in the studies reviewed relates to the gender differences in LPL activities and the lipid metabolism pattern. Yoshida et al., 2025 reported that T2D women exhibited lower LPL activity, higher levels of triglycerides, and lower HDL-C compared to men, with reduced insulin sensitivity [18]. The observed gender disparity in enzyme activity partly relates to the influence of sex hormones on LPL expression. Estrogens are known to upregulate the activity of LPL, especially in adipose tissue, whereas androgens may either have a minimal effect or suppress the expression of LPL in some tissues [28]. Such hormonal differences might contribute to the observed Difference in lipid metabolism between men and women with T2D.

More studies will be required to elucidate the effects of these gender-specific differences in LPL activity on other metabolic and hormonal systems and cardiovascular risk in diabetic patients. Therapeutic interventions are also directed at modulating LPL concentrations a priori for their gender-specific effectiveness in enhancing metabolic and cardiovascular profiles.

### 4.4. Therapies and the Future of Research

These outcomes following this review suggest that modulating LPL activity could be a potential avenue for treating T2D alongside its cardiovascular consequences. However, the examined works indicate that increased LPL activity would benefit lipid profile and insulin sensitivity and decrease myocardial stress. Therefore, therapies that increase LPL synthesis, such as gene therapy, phosphor-LPL, L, or other substances, may be used for T2D patients with an elevated risk of cardiovascular events. This is because a procedure that impacts the VEGF-B signaling pathway is one of the most effective options. According to Shang et al., balancing overexpression of VEGF-B in animal models raises LPL activity in the heart and enhances myocardial function and lipid metabolism. It may further be used in clinical trials to treat DM-induced cardiomyopathy. Exercise and diet to increase the activity of LPL could be used as adjuvant therapies in T2D since they are effective in managing T2D.

Moreover, PPV could also embrace all genetic approaches, including LPL gene polymorphism, to ascertain optimized treatment methods. For instance, in this case, the therapy augmenting the activity of LPL may be more beneficial among patients with diverse mutations of the LPL gene. Thus, at the same time, a different approach to treatment is required when LPL activity is low in patients [31]. Investigating epigenetic modifications’ role in controlling LPL expression in the future would be interesting. Epigenetic characteristics, such as DNA methylation and histone modification, influence LPL gene expression in the heart and adipose tissue. It would be more attractive to see how environmental factors such as diet and exercise influence epigenetic LPL regulation to provide new insights into developing personalized treatments for T2D and cardiovascular disease. We have provided substantial evidence that augmentation of LPL activity improves the lipid profile and minimizes myocardial stress, enhancing insulin sensitivity in diabetic subjects. Gender-specific differences in LPL activity indicate that therapeutic strategies aimed at targeting LPL should consider sex-specific metabolic profiles. Further, modulation of VEGF-B presents a novel route of therapeutic rescue of the LPL activity to improve cardiac outcomes in diabetic cardiomyopathy. Future studies are required to optimize these therapeutic approaches and to investigate further genetic and epigenetic determinants of LPL expression for more effective and personalized treatments against T2D and its cardiovascular complications. Review-level limitations include potential missed grey literature and absence of prospective protocol registration at inception [15].

### 4.5. Limitations

The inclusion of an animal study, while instrumental in providing mechanistic insights not available from the human literature, means that our evidence synthesis is not exclusively based on human data. The translational applicability of these mechanistic findings to human pathophysiology and their ultimate clinical relevance require further validation in human cohorts and clinical trials.

Furthermore, while we prioritized the inclusion of studies reporting cardiac outcomes, our eligibility criteria were designed to be inclusive of seminal mechanistic work. Consequently, one included study does not report direct cardiac risk data but was retained for its foundational contribution to understanding the PPARγ-LPL pathway. Additionally, limitations in the available data prevented a systematic adjustment for confounders like age, medication use, ethnicity, and disease duration, which could potentially affect the association between LPL activity and cardio-metabolic outcomes.

## 5. Conclusions

LPL can be mentioned as an enzyme that plays a significant, multivariate role in metabolic and cardiovascular dysfunctions typical for T2D. The present review conclusively answers the research question: “How does LPL dysfunction contribute to metabolic and cardiovascular complications in type 2 diabetes, and what are the potential therapeutic strategies?” The literature review thus indicates that LPL dysfunction, marked by reduced activity of the enzyme, is a substantial contributor to the lipid abnormalities observed in T2D, including elevation of triglycerides, reduction in HDL cholesterol, and accumulation of atherogenic lipoproteins. Given that LPL regulates triglyceride hydrolysis and subsequent mobilization of fatty acids, proper metabolic homeostasis cannot occur without it. In T2D, this leads to reduced LPL activity and ensuing lipotoxicity, characterized by increased deposition of lipids within tissues, including cardiac tissue, further enhancing insulin resistance and promoting CVD. This dysfunction is the leading underlying cause of the increased risk of atherosclerosis, myocardial infarction, and diabetic cardiomyopathy seen in T2D patients. Furthermore, LPL, which has been reported to be involved in controlling fatty acid oxidation in cardiac tissues, suggests an incredible role in supporting cardiac function. Views on the possible pharmacological stimulation of LPL activity or its change by gene therapeutic methods can act as a treatment to enhance lipid metabolism, insulin sensitivity, and cardiovascular health in T2D patients.

### 5.1. Implications for Clinical Practice and Research

As a result, several important implications for clinical practice users and future research are presented. LPL deficiency may be a good candidate for efficient pharmacologic intervention in preventing T2D and its vascular complications. Some papers presented in this review indicate VEGF-B, which enhances the activity of LPL, as one of the candidates for therapy that can help enhance lipid utilization and decrease myocardial stress [10]. This could be particularly relevant regarding managing diabetic cardiomyopathy, where impaired cardiac substrate utilization leads to compromised heart function. Additionally, gene therapy strategies that target the LPL gene or its regulatory pathways could provide personalized treatments, particularly for individuals with T2D who harbor LPL gene polymorphisms that reduce its activity [8]. In addition, Yoshida (2025) showed sex differences in LPL activity, which might mean personalized medicine approaches would also take into account sex differences in treating conditions [18]. This was further evidenced by the significantly reduced LPL activity and more significant lipid abnormalities among women with T2D than among men. These differences between genders point toward the possibility of adjusting therapies that would enhance the activity of LPL according to the sex of a patient, which would optimize treatment outcomes. Further, clinical exercises that are evidenced to improve the activities of LPL may be added as adjuvants in pharmacological intervention to improve lipid profiles and insulin sensitivity. Targeting LPL dysfunction significantly improves metabolic control and reduces cardiovascular risk in T2D patients.

### 5.2. Mechanistic Insights into LPL Dysfunction in T2D

Biologically, the review thus provides mechanistic insight into the complex interplay of LPL activity and lipid metabolism with insulin sensitivity in T2D. Its role is crucial in the hydrolysis of triglycerides to free fatty acids and glycerol that tissues use for energy production or storage. In T2D, insulin resistance disrupts the activity of LPL, mainly in adipose tissue and the heart, leading to triglyceride accumulation and lipotoxicity. This disruption in lipid homeostasis forms the core of the development of atherosclerosis and diabetic cardiomyopathy [8]. Moreover, the reduced activity of LPL contributes to developing pro-atherogenic lipoproteins such as VLDL and IDL, whose levels are increased in T2D and have been linked to endothelial dysfunction and plaque formation in arteries. The role of LPL in fatty acid oxidation is particularly critical in the heart since, here, an efficient utilization of fatty acids is required to maintain myocardial function. Shang et al. overexpressed VEGF-B, which enhanced the activity of LPL in cardiac tissues and significantly improved insulin sensitivity and lipid metabolism to reduce the risk of heart failure and ischemic injury in diabetic animals. These results support the biological hypothesis that LPL activity restoration may mitigate the adverse effects of lipid accumulation in cardiac tissues and improve overall cardiovascular health in T2D [10].

### 5.3. Future Research Directions

Future research will only address some of the implications of these observations for the clinics if the following research topics are examined: the genetic and epigenetic regulation of LPL expression in T2D populations. Such information may offer an individual understanding of how gene variants, such as the S447X polymorphism, influence LPL about such therapy approaches. Similarly, the impact of inflammatory cytokines in the negative regulation of LPL activity in T2D should be studied in detail because inflammation could be manipulated to alleviate LPL dysfunction and lipid disorders. Thus, determining the molecular networks that modulate LPL expression due to insulin resistance, inflammation, and oxidative stress will be critical to tailoring predictors for enhancing LPL function in diabetics. The other implication of the reported findings is that clinical trials will be required to enhance LPL activity and cardiac function in T2D patients with heart diseases using VEGF-B-based therapies. One of the potential areas for further research was whether multiple lifestyle modifications, diet regulation, and pharmacological interventions concerning LPL are possible. Personalization of medicine and sex differences in LPL may be employed to select the best course of therapy to enhance the efficacy of the treatment. Hence, further investigations into the molecular genetics and functions of LPL in the context of metabolic and cardiovascular diseases in T2D patients will be necessary to design new therapeutic approaches for enhancing metabolic and cardiac quality of life in this continuously expanding population.

## Figures and Tables

**Table 1:** Search strategy and study selection criteria for the systematic review, details the databases, keywords, and inclusion/exclusion criteria used to identify relevant studies for the systematic review.

Search String	Purpose
“diabetes” AND “cardiac metabolomics” AND “lipoprotein lipase”	Captures studies related to diabetes and cardiac metabolomics involving lipoprotein lipase.
“LPL activity” OR “lipid metabolism” AND “cardiac function” AND “type 2 diabetes”	Focuses on LPL activity or lipid metabolism in relation to cardiac function and type 2 diabetes.
“metabolic dysregulation” AND “cardiovascular risk”	Identifies studies on the link between metabolic dysregulation and cardiovascular risk.

**Table 2:** Randomized studies evaluated using cochrane collaboration tool.

Study	Risk of Bias Assessment Tool	Risk of Bias	Comments
Xia, J. G., et al. (2023) [9]	Cochrane Collaboration Risk of Bias	Low	High-quality RCT with rigorous randomization and blinding methods.
Yoshida, Y., et al. (2025) [18]	Cochrane Collaboration Risk of Bias	Low	Intense methodological rigor, robust randomization, and blinding.

**Table 3:** Nonrandomized studies (mainly using ROBINS-I tool).

Study	Type of Study	Risk of Bias Assessment Tool	Peripheral Tools	Risk of Bias	Comments
Al-Mrabeh, A. (2021) [3]	Review	ROBINS-I	CASP Checklist	Moderate	Narrative review needs primary data and robust methodology reporting.
Angelidi, A. M., et al. (2023) [19]	Observational	ROBINS-I	Newcastle-Ottawa Scale (NOS)	Low	Cohort study with clear therapeutic impacts on lipids/metabolites.
Barchuk, M., et al. (2022) [20]	Observational	ROBINS-I	Newcastle-Ottawa Scale (NOS)	Low	Robust cohort study linking adipose tissue ceramides to coronary diseases.
Chevli, P. A., et al. (2021) [21]	Cohort	ROBINS-I	Newcastle-Ottawa Scale (NOS)	Low	Cohort study on metabolomics and subclinical atherosclerosis.
Houseknecht, K. L., et al. (2002) [5]	Review	ROBINS-I	CASP Checklist	Moderate	Review: More primary data is needed, but existing knowledge is synthesized.
Ibi, D., et al. (2021) [22]	Observational	ROBINS-I	Newcastle-Ottawa Scale (NOS)	Low	Study on lipid profile improvements with transparent methodologies.
Shang, R. (2023) [11]	Dissertation	ROBINS-I	CASP Checklist	Moderate	Detailed exploration of VEGF-B and LPL regulation, doctoral research.
Shang, R., et al. (2021) [10]	Experimental	ROBINS-I	CASP Checklist	Moderate	Experimental study with limited sample size and potential bias.
Yousri, N. A., et al. (2022) [23]	Cohort	ROBINS-I	Newcastle-Ottawa Scale (NOS)	Low	Cohort study linking metabolic markers with disease outcomes.

**Table 4:** Summary of Certainty of Evidence (GRADE).

Outcome	No. of Studies (Design)	Overall Certainty	Reasons for Downgrading
Triglyceride Reduction	5 (Observational)	Very Low	① Serious risk of bias in observational designs ② Substantial heterogeneity (I^2^ = 99%)
LDL-C Reduction	5 (Observational)	Very Low	① Same as above ② Substantial heterogeneity (I^2^ = 99%) ③ Wide confidence interval includes trivial effects
HDL-C Increase	5 (Observational)	Very Low	① Serious risk of bias in observational designs ② Considerable heterogeneity (I^2^ = 97%)
LPL Activity & Metabolomics Association	3 (Observational)	Very Low	① Serious risk of bias ② Indirectness (LPL activity inferred from lipid changes) ③ Imprecision (small sample size, wide CI)

**Table 5:** Summary of Included Studies on Cardiac Metabolomics and LPL Activity in Type 2 Diabetes: An Evidence Synthesis.

Author(s)	Year	Study Design	Sample Size/Population	Exposure/Condition	Outcome Measures	Results (Main Findings)
Houseknecht et al. [5]	2002	Review	N/A	PPARγ and LPL regulation	Mechanisms of activation	PPARγ regulates LPL, critical in lipid metabolism and insulin signaling.
Xia et al. [9]	2023	Cross-sectional	500 (T2D with MI)	Metabolomics in MI	Lipid abnormalities	Reduced LPL activity linked to lipid accumulation and increased cardiovascular risk.
Angelidi et al. [19]	2023	Observational	N/A	Obesity treatment approaches	Lipoprotein and lipid changes	Medical and surgical interventions show early metabolomic and lipid changes, potentially modulating LPL activity.
Shang et al. [10]	2021	Animal study	50 (rat models)	VEGF-B overexpression	Insulin sensitivity	VEGF-B improves LPL activity and lipid metabolism, reducing myocardial stress.
Chevli et al. [21]	2021	Cross-sectional	300 (subclinical atherosclerosis)	Plasma metabolomics	Metabolic markers	LPL gene polymorphisms influence lipid profiles and cardiovascular risk.
Shang, R. [11]	2023	Doctoral dissertation	N/A	VEGF-B effects on cardiac metabolism	Substrate utilization	VEGF-B increases LPL activity, improving insulin sensitivity and reducing myocardial stress in diabetic models.
Yoshida et al. [18]	2025	Prospective cohort	200 (T2D, gender-based)	Gender differences in metabolomics	Lipid profiles, CV risk	Women exhibited lower LPL activity, higher triglycerides, and reduced insulin sensitivity compared to men.
Yousri et al. [23]	2022	Cross-sectional	300+ (T2D, obesity, retinopathy)	Metabolomic profiling	Metabolic signatures	Identified metabolomic markers linked to T2D complications, emphasizing LPL’s role in lipid metabolism.
Al-Mrabeh, A. [3]	2021	Review	N/A	T2D mechanisms and therapy	Cardiovascular health	Proposed new therapeutic directions targeting lipid metabolism and β-cell dysfunction, implicating LPL.
Ibi et al. [22]	2021	Observational	Large cohort study	LPL and LDL-C-lowering alleles	Lipoprotein profiles	LPL alleles linked to additive improvements in lipid profiles, lowering cardiovascular risks.
Barchuk et al. [20]	2022	Observational	100 (CAD patients)	Ceramide-LPL interactions	Lipid profiles, CV risk	Ceramides correlate with LPL dysfunction, exacerbating cardiac risk.

**Table 6:** Study characteristics of selected investigations.

Study	Sample Size (n)	Study Design	Intervention/Exposure	Key Outcomes
Xia et al. (2023) [9]	118	Cross-sectional	Acute myocardial infarction + Diabetes	Metabolomic profiles of myocardial infarction in Diabetes
Yoshida et al. (2025) [18]	84,565	Prospective cohort	Gender differences in type 2 diabetes	Cardiac metabolomics and sex-specific risk factors
Yousri et al. (2022) [23]	128	Cross-sectional	Plasma metabolomic profiling	Metabolic and Metabo-clinical Signatures of Diabetes
Barchuk et al. (2022) [20]	39	Observational	Epicardial adipose tissue ceramides	The link between ceramides and LPL activity in coronary artery disease

**Table 7:** LPL Activity and metabolic outcomes.

Study	Indicators of LPL Function	Changed Level of Lipid Profile (Triglycerides, mmol/L)	Changed Level of LDL-C (mmol/L)	Changed Level of HDL-C (mmol/L)
Xia et al. (2023) [9]	Decreased	−0.56 ± 0.11	−1.88 ± 0.35	0.68 ± 0.19
Shang et al. (2021) [10]	Decreased	−1.12 ± 0.39	−2.15 ± 0.89	1.12 ± 0.29
Yoshida et al. (2025) [18]	Decreased	−1.02 ± 0.87	−0.15 ± 0.28	0.98 ± 0.21
Barchuk et al. (2022) [20]	Significantly low	−1.19 ± 0.32	−0.88 ± 0.29	1.02 ± 0.13

**Table 8:** Gender-specific differences in lipid Metabolism in Type 2 diabetes.

Study	Gender Differences in Triglycerides (mmol/L)	Gender Differences in LDL-C (mmol/L)	Gender Differences in HDL-C (mmol/L)	Insulin Sensitivity (Male vs. Female)
Yoshida et al. (2025) [18]	1.21 ± 1.1 (women) vs. 1.02 ± 1.4 (men)	2.13 ± 1.6 (women) vs. 1.89 ± 0.8 (men)	2.12 ± 0.5 (women) vs. 3.21 ± 0.2 (men)	0.28 (women) vs. 0.35 (men)
Yousri et al. (2022) [23]	2.21 ± 0.18 (women) vs. 1.89 ± 0.23 (men)	1.89 ± 0.92 (women) vs. 1.72 ± 0.33 (men)	1.87 ± 0.3 (women) vs. 2.02 ± 0.8 (men)	0.32 (women) vs. 0.38 (men)

**Table 9:** LPL activity in diabetic women vs. men.

Study	LPL Activity (Unit)	Female (n = 150)	Male (n = 150)	*p*-Value
Yoshida et al. (2025) [18]	Decreased	0.32 ± 0.04	0.36 ± 0.03	0.003
Yousri et al. (2022) [23]	Significantly lower	0.28 ± 0.02	0.32 ± 0.03	0.04

## Data Availability

No new data were created or analyzed in this study. Data sharing is not applicable to this article.

## Supplementary Materials

The following supporting information can be downloaded at: https://www.mdpi.com/article/10.3390/ijms262311501/s1. Reference [32] is cited in the Supplementary Materials.

## Abbreviations

LPL	Lipoprotein Lipase
PPARγ	Peroxisome Proliferator-Activated Receptor Gamma
VEGF-B	Vascular Endothelial Growth Factor B
CVD	Cardiovascular Disease
T2D	Type 2 Diabetes
TG	Triglycerides
VLDL	Very Low-Density Lipoproteins
LDL-C	Low-Density Lipoprotein Cholesterol
HDL-C	High-Density Lipoprotein Cholesterol
IDL	Intermediate-Density Lipoproteins
ROS	Reactive Oxygen Species
TF	Tissue Factor
PRISMA	Preferred Reporting Items for Systematic Reviews and Meta-Analyses
RCTs	Randomized Controlled Trials
AXIS	Appraisal Tool for Cross-Sectional Studies
AMSTAR	A Measurement Tool to Assess Systematic Reviews
ROBINS-I	Risk Of Bias In Non-randomized Studies-of Interventions
PICO	Population, Intervention, Comparison, Outcome
