# Supplementary material for: Cardiac Metabolomic Alterations in Diabetes: Interplay with Lipoprotein Lipase—A Systematic Review
Source: Int J Mol Sci. 2025 Nov 27;26(23):11501. doi: 10.3390/ijms262311501 (PMC12692324; doi:10.3390/ijms262311501)
Supplement: Supplementary file 1 [file ijms-26-11501-s001.zip › ijms-3941923 - Supplementary Material .pdf]

Table S1. Full Search Strategies. This document provides the complete and reproducible search strategies for all databases searched in this systematic review. Searches were conducted from 2000 to 2024, with the final search executed on [26 November 2024]. The search strategy was built around key concepts of "Type 2 Diabetes," "Cardiac Metabolomics," and "Lipoprotein Lipase." Boolean operators (AND, OR) and truncation (*) were used to broaden the search.

**EMBASE**	"diabetes" AND "cardiac metabolomics" AND" lipoprotein lipase"<br>"LPL activity" OR "lipid metabolism" AND "cardiac function" AND "type 2 diabetes"<br>"metabolic dysregulation" AND "cardiovascular risk"<br>Date Searched: [22 August 2025]<br>Limits/Filters: Publication years 2000-2024
**PsycINFO**	"diabetes" AND "cardiac metabolomics" AND" lipoprotein lipase"<br>"LPL activity" OR "lipid metabolism" AND "cardiac function" AND "type 2 diabetes"<br>"metabolic dysregulation" AND "cardiovascular risk"<br>Date Searched: [22 August 2025]<br>Limits/Filters: Publication years 2000-2024
**AMED**	"diabetes" AND "cardiac metabolomics" AND" lipoprotein lipase"<br>"LPL activity" OR "lipid metabolism" AND "cardiac function" AND "type 2 diabetes"<br>"metabolic dysregulation" AND "cardiovascular risk"<br>Date Searched: [22 August 2025]<br>Limits/Filters: Publication years 2000-2024
**LILACS**	"diabetes" AND "cardiac metabolomics" AND" lipoprotein lipase"<br>"LPL activity" OR "lipid metabolism" AND "cardiac function" AND "type 2 diabetes"<br>"metabolic dysregulation" AND "cardiovascular risk"<br>Date Searched: [22 August 2025]<br>Limits/Filters: Publication years 2000-2024
**Web of Science**	"diabetes" AND "cardiac metabolomics" AND"lipoprotein lipase"<br>"LPL activity" OR "lipid metabolism" AND "cardiac function" AND "type 2 diabetes"<br>"metabolic dysregulation" AND "cardiovascular risk"<br>Date Searched: [22 August 2025]<br>Limits/Filters: Publication years 2000-2024

Table S2: Prespecified Rules for Handling Missing or Unclear Information This document outlines the rules and assumptions prespecified by the review team for handling missing, unclear, or unreported data during the data extraction and synthesis process. These rules were applied consistently across all included studies.

1. Data Extraction and Conversion Rules

1.1. Missing Summary Statistics (for Continuous Outcomes)

Missing Standard Deviation (SD):

1. Primary Approach: If not reported, SD will be calculated from other available measures (e.g., Standard Error (SE), confidence intervals (CIs)) using standard conversion formulas[1].
   - $SD = SE \times \sqrt{n}$
   - For 95% CI: $SD = \sqrt{n} \times (\text{Upper Limit - Lower Limit}) / 3.92$
2. Secondary Approach: If no data is available for calculation, we will contact the corresponding author of the study via email to request the missing data. Two reminder emails will be sent at two-week intervals.
3. Last Resort: If the data cannot be obtained, and the study is deemed critical to the synthesis, we will impute the SD using the following hierarchy:
   a. Use the average SD from other, most similar studies included in the same meta-analysis.
   b. If no similar studies are available, the study will be described narratively but not included in the meta-analysis.

- Missing Mean or Median:
  - If the mean is missing but the median is reported, we will not impute the mean from the median. The study will be included in a narrative synthesis but excluded from the meta-analysis of continuous outcomes.
  - If only a graph is presented, plot digitization software (e.g., WebPlotDigitizer) will be used to extract approximate values, and this will be clearly noted.

1.2. Data Presented in Other Formats

- Data reported as "Range": Will not be used to impute SD. The study will be excluded from meta-analysis.
- Data reported as "Interquartile Range (IQR)": If the sample size is large (>50) and the distribution is assumed to be approximately normal, we will estimate the SD as $IQR / 1.35$. This assumption will be clearly stated in the results.

2. Assumptions for Unclear Methodological Details

- "LPL Activity" Assay: If a study states that "LPL activity was measured" but does not specify the assay method (e.g., radioactive, fluorometric), it will be categorized as "Unspecified Assay" in the analysis. We will not assume a specific method.
- "Cardiac Tissue" Specificity: If a metabolomic measurement is described as being from "cardiac tissue" without specifying the exact type (e.g., left ventricle, atrium), it will be assumed to represent a general myocardial sample. This will be considered a potential source of heterogeneity.
- Study Design: If the study design is not explicitly stated (e.g., "prospective study"), it will be classified based on the described methodology. If this is unclear, it will be categorized as an "Observational Study, design unspecified".

3. Rules for Handling "Missing" Studies

- Unobtainable Full Texts: If a study identified by abstract appears eligible but the full text cannot be retrieved through library services, inter-library loan, or by contacting authors, it will be listed under "Studies awaiting classification" and noted as a potential limitation.
- Studies with Overlapping Populations: If multiple publications report on the same or overlapping cohort:
  1. We will contact the authors for clarification on unique participants.
  2. If no response is received, we will include the publication with the largest sample size or the most complete outcome data to avoid double-counting.

4. Outcome-Specific Rules

- Lipid Measurements (TG, LDL-C, HDL-C): All values will be converted to mg/dL for consistency. Conversion factor: 1 mmol/L triglycerides ≈ 88.57 mg/dL; 1 mmol/L cholesterol ≈ 38.67 mg/dL.
- Metabolomic Markers (e.g., Acylcarnitines, Ceramides): If concentrations are reported in different units (e.g., nmol/g vs. μmol/L), they will not be pooled in a meta-analysis. Instead, the direction and significance of effects will be synthesized narratively.

5. Risk of Bias Assessment

- Unclear sequence generation or allocation concealment in RCTs will be judged as having "Some concerns" in the RoB 2 tool.
- Unadjusted confounding in observational studies will be judged as contributing to a "Serious" risk of bias in the ROBINS-I tool.

Table S3. Summary of Findings (GRADE): Effects of LPL Modulation on Lipid Metabolism and Cardiac Risk in Patients with Type 2 Diabetes.

Outcome	Certainty of Evidence	Absolute Effect (95% CI)	Participants (Studies)
Triglyceride levels	Very Low a, b, c	MD -0.76 mmol/L (-1.31 to -0.85)	541 (4)
LDL-C levels	Very Low a, b, c, d	MD -1.27 mmol/L (-2.89 to 0.21)	541 (4)
HDL-C levels	Very Low a, b, c	MD 0.20 mmol/L (0.17 to 0.23)	541 (4)

Table S4. Risk of Bias Assessment for Included Studies.

Part A: Risk of Bias for Randomized Controlled Trials (Assessed using the Cochrane RoB 2 tool)						
**Study**	**Randomization Process**	**Deviations from Intended Interventions**	**Missing Outcome Data**	**Measurement of the Outcome**	**Selection of the Reported Result**	**Overall Bias**
Xia et al. (2023) [9]	Low	Low	Low	Low	Low	Low
Yoshida et al. (2024) [12]	Low	Low	Low	Low	Low	Low

Part B: Risk of Bias for Non-Randomized Studies (Assessed using the ROBINS-I tool)								
Study	**Confounding**	**Participant Selection**	**Intervention Classification**	**Deviations from Interventions**	**Missing Data**	**Outcome Measurement**	**Selective Reporting**	**Overall Bias**
Angelidi, A. M., et al. (2023) [19]	Low	Low	Low	Low	Low	Low	Low	Low
Barchuk, M., et al. (2022) [20]	Low	Low	Low	Low	Low	Low	Low	Low
Chevli, P. A., et al. (2021) [21]	Low	Low	Low	Low	Low	Low	Low	Low
Ibi, D., et al. (2021) [22]	Low	Low	Low	Low	Low	Low	Low	Low
Yousri, N. A., et al. (2022) [23]	Low	Low	Low	Low	Low	Low	Low	Low
Al-Mrabeh, A. (2021) [3]	Moderate	Moderate	Low	Low	Moderate	Moderate	Moderate	Moderate
Houseknecht, K. L., et al. (2002) [5]	Moderate	Moderate	Low	Low	Moderate	Moderate	Moderate	Moderate
Shang, R. (2023) [11]	Moderate	Moderate	Low	Low	Moderate	Moderate	Moderate	Moderate
Shang, R., et al. (2021) [10]	Moderate	Moderate	Low	Moderate	Moderate	Moderate	Moderate	Moderate
